# Specialist Palliative Care Service Involvement and Prognostic Factors in Patients Presenting With Acute Limb Ischaemia to Waikato Hospital, Hamilton, New Zealand: A Retrospective Review

**DOI:** 10.1155/ijvm/8163761

**Published:** 2026-10-02

**Authors:** Stacey Hooper, Stephanie Eckoldt, Stuart Brown, Jun Cheul Park, Manar Khashram

**Affiliations:** ^1^ Department of Palliative Medicine Waikato Hospital Hamilton New Zealand waikatodhb.health.nz; ^2^ Department of Vascular Surgery Waikato Hospital Hamilton New Zealand waikatodhb.health.nz

## Abstract

**Background:**

There is significant morbidity and mortality associated with acute limb ischaemia (ALI) and prognostication is complex. Current literature reports underutilisation of specialist palliative care (SPC) and lack of prognostic indicators to guide decision‐making and referral.

**Objective/Aim:**

The aim of this study is to review and analyse demographic, clinical and mortality data for a cohort of ALI patients to determine potential prognostic indicators, SPC referral patterns and SPC services received.

**Method:**

Retrospective clinical record reviews for a cohort of ALI patients referred to the Waikato Hospital Vascular Surgery team in a specified time period. This included laboratory, imaging and death documentation and functional status at baseline and ALI diagnosis.

**Results:**

One hundred fifty‐five ALI patients were identified of whom 140 met the inclusion criteria. A 30‐day mortality was 19% and 1‐year mortality was 31% for the study cohort. Lactate > 2.2 mmol//L, albumin < 27 g/L Rutherford III staging and reduced functional capacity (Australia‐modified Karnofsky Performance Scale score ≤ 50 at baseline and ≤ 40 at ALI diagnosis) were found to be independent predictors of mortality. Referral for SPC was 14% of the study cohort, usually late in the admission and often in the last few days of life. SPC provided a wide range of services, frequently exceeding the referral request.

**Conclusion:**

The four independent predictors of mortality could be used to identify ALI patients with a poor prognosis, encourage earlier referral for SPC, support appropriate symptom management and enable discussions on patient priorities.

## 1. Introduction

ALI is defined as a sudden compromise of the arterial blood circulation to a limb with associated symptom onset of less than 2 weeks [1, 2]. The annual incidence of ALI has been estimated at 10–26 of 100,000 people [3]. ALI is an advanced form of peripheral arterial disease (PAD) with considerable morbidity and mortality [1, 4]. Literature review found one‐year amputation rates of 15%–20%, one‐year mortality of 15%–40% and 5‐year mortality exceeding 50% [4]. Studies utilising early revascularisation reported 30‐day mortality and amputation rates of 10%–15% [5, 6]. Annual mortality rates after amputation were up to 48% [7], matching 1‐year mortality rates for stage IV colon cancer [8] and surpassing that for New York Heart Association class 4 congestive heart failure (30%) [9] and acute exacerbation of chronic obstructive pulmonary disease (23%) [10].

The benefits of SPC involvement for symptom management, quality of life and alignment of treatment options with goals of care for patients with cancer, chronic obstructive pulmonary disease and congestive heart failure have been described [11, 12]. Despite the high morbidity and mortality seen with ALI, referral for SPC is limited and frequently late in the disease process [13–15]. A 2021 retrospective, single‐centre study by Kwong et al. [16] found inadequate provision of SPC for patients with advanced PAD and, when consultation did occur, death often occurred before amputation was undertaken. Thus, the benefit of early assessment for symptom management and goals of care discussions was lost.

Rutherford classification of acute limb ischaemia is commonly utilised for early staging of ALI severity by assigning patients to one of four broad categories (Table 1) [17]. The stage affects viability and treatment options. Treatment for severe PAD depends on the degree of tissue damage and potential for limb preservation. Maintaining function is the primary goal [1, 2]. Management of ALI involves restoration of blood flow to the limb using vasodilating medications or surgical bypass procedures [1, 2]. If the limb is not viable, the alternatives are amputation or conservative care [1, 2]. Management of patients′ pain can be difficult and complex. The challenge lies in determining the appropriate intervention in the context of an unclear prognosis.

**Table 1:** Rutherford classification of ALI [17].

Rutherford staging	Description
I	Viable—not immediately threatened
IIa	Threatened: marginally—salvageable if promptly treated
IIb	Threatened: immediately—salvageable with immediate revascularisation
III	Irreversible—major tissue loss or permanent nerve damage inevitable

There has been little research on identifying ALI patients with a poor prognosis at diagnosis. In 2015, Howard et al. [18] found male gender, renal impairment and a history of previous coronary artery disease were independently associated with 30‐day mortality and amputation, whereas renal impairment and age were associated with increased 1‐year mortality. Kulezic and Acosta [19] found gender, age, atrial fibrillation, Rutherford stage > IIb and anaemia increased the risk of major amputation and/or death at 1 year. Further research is needed to determine prognostic factors and develop guidelines for planning the most beneficial management of ALI patients from initial presentation.

Prognosis is often determined using multiple factors including a functional assessment. In palliative medicine, the Australia‐modified Karnofsky Performance Scale (AKPS) (Table 2) was developed by amalgamating the original Karnofsky Performance Status (KPS) and Thorne‐modified Karnofsky Performance Status (TKPS) enabling assessment of patients′ functional performance within diverse care settings [20]. Abernethy et al. [20] compared these three tools in palliative patients and found the AKPS to be superior, particularly in predicting survival outcomes at the lower ranges of the scale. More recently, Blum et al. [21] reported significant differences in patient functional performance at time of referral for SPC for many serious illnesses and suggested that AKPS scores could be helpful in identifying opportunities for earlier referral. In another study, O′Connor et al. [22] found patients spent approximately 24 days with an AKPS of 20 before declining and dying. These studies suggest AKPS may be a useful tool to help guide prognosis and treatment decisions in patients presenting with ALI.

**Table 2:** The Australia‐modified Karnofsky Performance Scale (AKPS) [20].

AKPS assessment criteria	Score
Normal; no complaints; no evidence of disease	100
Able to carry on normal activity; minor sign of symptoms of disease	90
Normal activity with effort; some signs or symptoms of disease	80
Cares for self; unable to carry on normal activity or to do active work	70
Able to care for most needs; but requires occasional assistance	60
Considerable assistance and frequent medical care required	50
In bed more than 50% of the time	40
Almost completely bedfast	30
Totally bedfast and requiring extensive nursing care by professionals and/or family	20
Comatose or barely rousable	10
Dead	0

### 1.1. Aims of the Research

The study′s aim was to determine prognostic factors that could be used to highlight ALI patients with poor prognosis, and optimise their treatment and timely referral for SPC. In addition, to investigate SPC referral patterns and what services the SPC service had provided for patients.

## 2. Methods

### 2.1. Design

The study was a retrospective observational single‐centre study of inpatients with ALI who were referred to the vascular service at Waikato Hospital, a New Zealand tertiary level hospital, between 1 January 2019 and 31 May 2023.

### 2.2. Patient Selection

Patients were identified using the Australian Vascular Audit application, a database that includes all vascular referrals. ALI was defined as a sudden compromise of the arterial blood circulation to a limb with associated symptom onset of less than 2 weeks [1, 4].

### 2.3. Inclusion and Exclusion Criteria

Patients were included in the study cohort if they were referred to the vascular service having either presented with ALI or developed ALI whilst in hospital for another problem (including as the result of a procedure). Patients were excluded if their ALI was the result of trauma, they had missing records, were not admitted or ALI was ruled out after vascular assessment.

### 2.4. Demographics and Comorbidities

Patient age, gender, ethnicity and comorbidities (see Table 3) were ascertained from their clinical records.

**Table 3:** Comorbidities documented with descriptions and/or definitions.

Comorbidity	Description/definition
Ischaemic heart disease (IHD)	Included previous myocardial infarction, angina, coronary artery bypass surgery or percutaneous stent insertion or listed in background medical history in medical documentation.
Congestive cardiac failure (CCF)	Listed in background medical history in medical documentation.
Atrial fibrillation (AF)	Listed in background medical history in medical documentation.
Hypertension (HTN)	Defined as a systolic blood pressure > 140, diastolic blood pressure > 90 or listed in background medical history in medical documentation.
Chronic kidney disease (CKD)	Defined as CKD 3+ (estimated glomerular filtration rate 45‐59).
Cerebrovascular accident (CVA)	Included transient ischaemic attack, ischaemic or haemorrhagic stroke, subdural haemorrhage, subarachnoid haemorrhage and brain metastatic disease.
Peripheral vascular disease (PVD)	Listed in background medical history in medical documentation.
Chronic liver disease (CLD)	Listed in background medical history in medical documentation.
Pulmonary disease	Included chronic conditions such as asthma, chronic obstructive pulmonary disease, bronchiectasis, lung cancer and tuberculosis.
Smoking status	Current, ex‐smoker or never smoked cigarettes.
Malignant disease	Listed in background medical history in medical documentation.
Diabetes	Listed in background medical history in medical documentation.
Rheumatologic disease	Included chronic conditions such as rheumatoid arthritis.
Dementia	Listed in background medical history in medical documentation.

### 2.5. Medical Assessment

Patient data were collected from paper and digital (Clinical Workstation [CWS], Midland Clinical Portal, Pathlab Portal and Death Documents) records. Laboratory data included albumin, lactate and C‐reactive protein (CRP) levels. Functional status at baseline (preadmission), at ALI diagnosis and on discharge was obtained from the self‐reported AKPS assessment completed by all patients on admission to Waikato Hospital, or from formal functional assessments by occupational therapy (OT) or physiotherapy (PT).

A vascular surgery registrar extracted the data on patients′ vascular pathology, severity of disease (using Rutherford classification) and treatment from the Australian Vascular Audit application and their clinical records. Treatment was divided into two categories:

1. Surgical—including endovascular revascularisation procedures and amputation.
2. Conservative—involving nonsurgical symptom management, including anticoagulation treatment.

Revascularisation was defined as restoration of blood flow to the affected limb by any means including both surgical and conservative (anticoagulation) treatments.

### 2.6. Specialist Palliative Care Service Involvement

ALI patients referred for SPC were identified by cross‐referencing the vascular database with the palliative care referral database. Their clinical records were reviewed by a palliative care advanced trainee who extracted the following data:

• The interval between diagnosis of ALI and death.
• The interval between diagnosis of ALI and referral for SPC.
• The interval between referral for SPC and death.
• Whether a referral for SPC was documented.
• The reason for SPC referral.
• What services were provided to patients by the SPC team?
• Whether SPC were involved in treatment option decisions (conservative versus surgical treatment).

The trainee also went through the clinical records of patients not referred for SPC who died within 30 days of ALI diagnosis to determine what SPC support could have been offered.

### 2.7. Outcomes

Patient outcomes included 30‐day mortality, 1‐year survival and overall survival at the end of the study period (3.5 years).

### 2.8. Statistical Analysis

Statistical analysis was performed using GraphPad Prism scientific software.

### 2.9. Ethics Statement

Waikato Hospital (RD022048) and the Te Puna Oranga Māori Research Review Committee provided ethical approval. Review by the Health and Disability Ethics Committees was not necessary.

## 3. Results

### 3.1. Demographics

The study cohort demographic data are summarised in Figure 1 and Table 4. Most patients were in their sixties or seventies, with a slight male predominance. No statistically significant difference was found in gender distribution between patients identifying as New Zealand European (NZE) or other European (*n* = 95) and Māori (*n* = 45) using the chi‐squared test (*p* = 0.39). Similarly, no statistically significant difference was identified in all‐cause mortality between NZEs and other Europeans and Māori (Figure 2), nor was Māori ethnicity found to be an independent predictor of mortality (*p* = 0.62).

**Figure 1:**
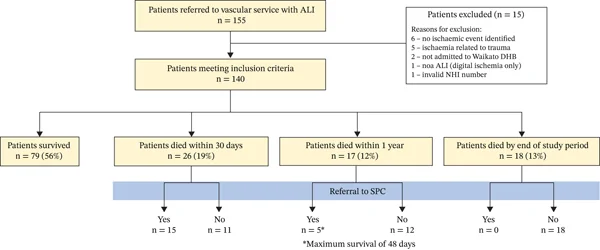
Study cohort.

**Table 4:** Demographic data.

Demographic	Detail	*n* (%)
Age	Mean: 72	
	Median: 74	
	Range: 29–92	
	SD: ±12.9	

Biological sex	Male	80 (57%)
	Female	60 (43%)

Ethnicity	Indian/Asian	3 (2%)
	Māori/Pacific peoples	46 (33%)
	NZ European/European	91 (65%)

Smoking status	Current smoker	35 (25%)
	Ex‐smoker	48 (34%)
	Nonsmoker	57 (41%)

**Figure 2:**
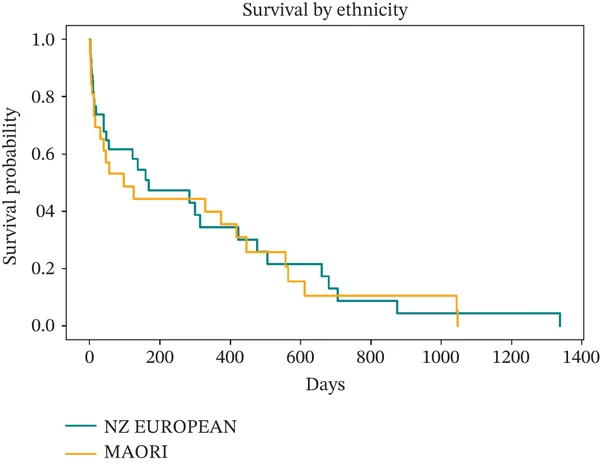
Survival by ethnicity.

### 3.2. Mortality

Mortality data can be seen in Table 5. Over the course of the study 61 (44%) of the patients died, 26 (19%) within 30 days of ALI diagnosis, 17 (12%) between 30 days and 1 year, and 18 (13%) after more than 1 year. Of the 26 patients that died within 30 days, 22 (85%) had Rutherford Stage IIb or III level ischaemia. Mortality increased with age, rising sharply in the 80–89 and 90+ age brackets groups. Overall survival is shown in Figure 3.

**Table 5:** Mortality data.

Measure	Detail	*N* (%)
Overall mortality (cumulative)	30 days	26 (19%)
	90 days	33 (15%)
	1 year	43 (31%)
	2 years	55 (39%)
	3 years	61 (44%)

Mortality by Rutherford (overall)	I	6/21 (29%)
	IIa	19/56 (34%)
	IIb	23/46 (50%)
	III	13/17 (77%)

Mortality by age	< 50	1/10 (10%)
	50–59	7511 (45.5%)
	60–69	11/31 (35.5%)
	70–79	17/46 (37%)
	80–89	22/36 (61.1%)
	90+	5/6 (83.3%)

**Figure 3:**
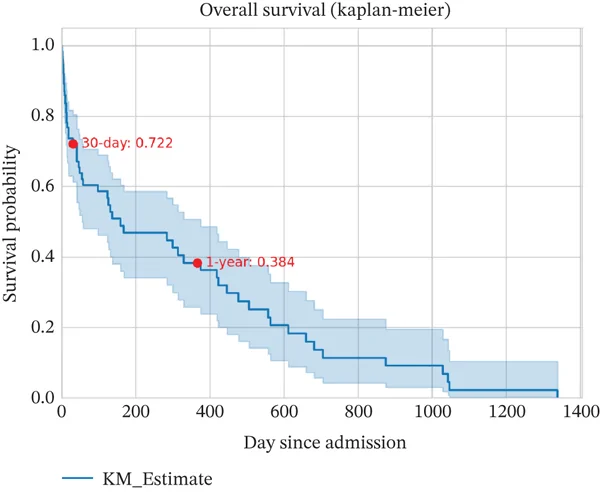
Overall survival.

Using a Cox model, mortality rose with Rutherford stage, and there was no significant change when adjusted by age (*p* = 0.80). Patients with Rutherford Stage III had an almost 10‐fold increased risk of death compared with patients with Rutherford Stage I (Hazard ratio [HR] ≈9.7 [95% CI 3.4–27.6], *p* ≈ 2 × 10^−5^). Rutherford Stage IIb patients also had an increased risk of death, but it was not statistically significant (HR ≈2.06, *p* = 0.12), and there was no difference between Rutherford Stages IIa and I.

The Kaplan–Meier survival analysis (Figure 4) demonstrated that patients presenting with Stage III Rutherford had the worst short‐term survival rates, followed by Rutherford IIb, IIa and I in descending order. Using a log‐rank test, higher Rutherford staging at ALI diagnosis was found to significantly predict mortality (*p* = 1.5 × 10^−7^), well below 0.05, indicating that Rutherford Stage III ischaemia was a significant prognostic marker for mortality in this cohort.

**Figure 4:**
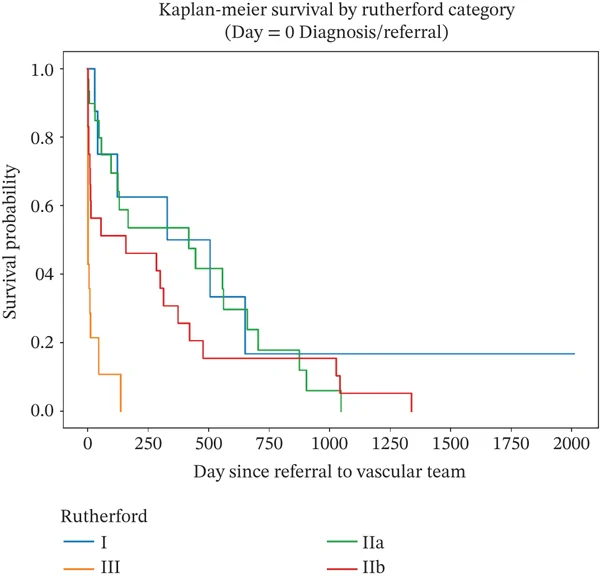
Kaplan–Meier survival curve by Rutherford stage.

### 3.3. Baseline Premorbid Status/Comorbidities

Cardiovascular disease (*n* = 93, 66%) and chronic kidney disease (*n* = 52, 37%) were the most common comorbidities in the study cohort (Table 6). Using multivariate (age, number of comorbidities and Rutherford stage) and univariate (number of comorbidities) models, HR of 1.05 and 1.01, and *p* values of 0.51–0.92 were found, indicating the effect was not statistically significant. Therefore, the number of comorbidities a patient presented with was not predictive of mortality.

**Table 6:** Premorbid status.

Measure	Detail	*n* = (%)
Major comorbidities	Cardiovascular disease	93 (66%)
	Chronic kidney disease (> Stage 3)	52 (37%)
	Previous diagnosis of PVD	43 (31%)
	Diabetes mellitus	42 (30%)
	Respiratory disease	33 (24%)
	Malignancy	31 (22%)
	Cerebrovascular disease	28 (20%)
	Dementia	12 (9%)
	Chronic liver disease	2 (1%)

Number of major comorbidities	Mean 2.2	
	Median 2	
	0	14 (10%)
	1	30 (21%)
	2	43 (31%)
	3	34 (24%)
	4	12 (9%)
	5	5 (4%)
	6	1 (0.7%)
	7	1 (0.7%)

AKPS at baseline	Mean 71	
	Median 70	
	SD ± 13	
	Mild functional impairment (80–100)	64 (46%)
	Moderate functional impairment (50–70)	73 (52%)
	Severe functional impairment (< 40)	2 (1%)

AKPS at ALI diagnosis	Mean 48	
	Median 50	
	SD ± 15	
	Mild functional impairment (80–100)	1 (0.5%)
	Moderate functional impairment (50–70)	89 (64%)
	Severe functional impairment (< 40)	49 (35%)

AKPS on discharge	Mean 51	
	Median 60	
	SD ± 25	
	Mild functional impairment (80–100)	13 (9%)
	Moderate functional impairment (50–70)	93 (67%)
	Severe functional impairment (< 40)	33 (24%)

### 3.4. Functional Status

At baseline almost all patients (*n* = 137, 98%) had an AKPS of 50 or more (Table 6). A Cox regression analysis and model of baseline AKPS showed that every one‐point increase was associated with a lower risk of death (approximately 4.4%; HR = 0.96 [95% CI 0.93–0.98], *p* = 0.0014). When adjusted for age, results were not significantly different (*p* = 0.40). Therefore, irrespective of age, patients with a higher functional capacity at baseline survived longer with a risk reduction of 0.956^{10}^ (approximately 0.63%). That is, there was a 37% lower risk of death for every 10‐point increase in AKPS.

Median score for AKPS at ALI diagnosis was 50, indicating that half of the cohort had moderate functional impairment. Using Cox regression analysis, AKPS at diagnosis was a slightly better predictor of mortality (HR per 10‐point increase = 0.785; *p* = 0.0446) than AKPS at baseline (HR per 10‐point increase = 0.71; *p* = 0.0411). There was a significant decline in AKPS from baseline to ALI diagnosis with a mean drop of 23.5 points. Table 7 shows the highest mortality rate of 80% with baseline AKPS of ≤ 50 and 79.6% with AKPS at ALI diagnosis of ≤ 40. Both AKPS measures when used alone are significant predictors of mortality (*p* < 0.001).

**Table 7:** Relationship between AKPS and mortality over the study period.

Baseline AKPS	Survival (%)

> 70	91.1%
51–70	40.8%
≤ 50	20%

AKPS at ALI diagnosis	Survival (%)

> 60	78.6%
41–60	74%
≤ 40	20.4%

At discharge the median AKPS was 60, suggesting improvement from functional status at diagnosis. Half of those with severe functional impairment died in hospital (AKPS 0 at discharge). Table 8 shows the mean functional status at baseline, diagnosis of ALI and on discharge for each Rutherford stage of ischaemia.

**Table 8:** Mean AKPS scores at baseline, diagnosis of ALI and on discharge at each Rutherford stage divided into survivors and nonsurvivors.

Rutherford stage	AKPS at baseline (mean)	AKPS on diagnosis (mean)	AKPS on discharge (mean)
I			
Survived	79	58	67
Died	63	45	52
IIa			
Survived	77	55	67
Died	65	44	42
IIb			
Survived	78	52	62
Died	63	39	33
III			
Survived	80	53	63
Died	50	30	0

### 3.5. Vascular Data

Vascular data in Table 9 shows a majority of patients had Rutherford Stage IIa (*n* = 56, 40%) or IIb (*n* = 46, 33%) at presentation. Revascularisation (including both surgical procedures and anticoagulation) was successful in 75% of patients, whereas the remaining 25% were not successful or had primary amputation treatment. A majority did not undergo major limb amputation.

**Table 9:** Vascular data.

	Detail	*n*(%)
Rutherford stage	I	21 (15%)
	IIa	56 (40%)
	IIb	46 (33%)
	III	17 (12%)

Revascularisation success	Yes	105 (75%)
	No	35 (25%)

Major limb amputation	Yes	10 (7%)
	No	130 (93%)

Those with a lower Rutherford stage, I or IIa, had higher rates of successful revascularisation (89% and 90%) compared with 70% for Rutherford IIb and 24% for Rutherford III. Lower rates of amputation were seen in Rutherford Stages I (0%) and IIa (3%) patients than those with Rutherford Stages IIb (11%) and III (18%).

### 3.6. Biomarker Data

To determine if biomarkers offered prognostic values, data on albumin, lactate and CRP was collected. Despite missing data for one‐third of the cohort (lactate values were unavailable for 42 patients and albumin values for 12), albumin and lactate levels showed a strong association with mortality, with high statistical significance. Those patients with albumin measurements showed HR ≈0.90 (*p* ≈ 6 × 10^−4^), and those with lactate measurements showed HR ≈1.85 (*p* ≈ 1.6 × 10^−5^).

The chi‐squared model was used to determine biomarker cut‐off values of significance (Figures 5 and 6). Data showed that patients with albumin values below 27 g/L^−1^ had a 3–4 times greater risk of mortality than patients with values greater than 27 g/L. Patients with lactate levels above 2.2 mmol/L^−1^ at admission consistently showed a 2–3 times increased mortality risk than those with lactate levels below 2.2 mmol/L. Cut‐off points were further validated using a cross‐validation model.

**Figure 5:**
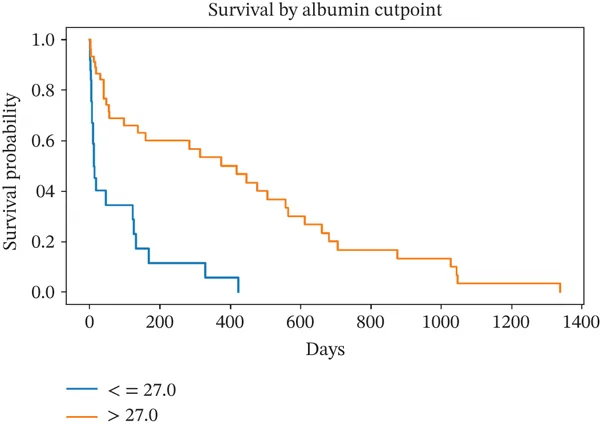
Albumin cut‐off value.

**Figure 6:**
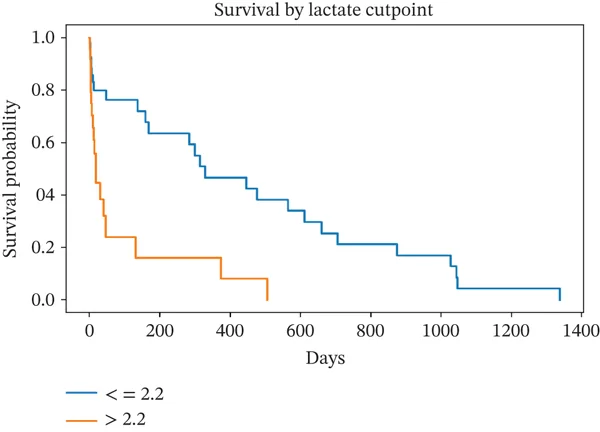
Lactate cut‐off value.

### 3.7. SPC Referral Data

Of the 140 patients reviewed, 20 (14%) were referred for SPC. Fifteen (75%) of these died within 30 days of ALI diagnosis with a maximum survival of 48 days for those referred. Of the 20 patients referred for SPC, 5 (20%) had unmet management needs that may have benefitted from earlier SPC referral. The average time from SPC referral to death was 13 days.

Nine patients (45%) were referred for SPC within 1 day of ALI diagnosis (Figure 7), whereas the other 11 (55%) were referred later, the latest being 3 weeks post ALI diagnosis. There were another 11 patients who died within 30 days of ALI diagnosis and were not referred for SPC despite having unmet management needs that may have benefitted from the service. Figure 8 shows potential support that the SPC team could have offered to these patients.

**Figure 7:**
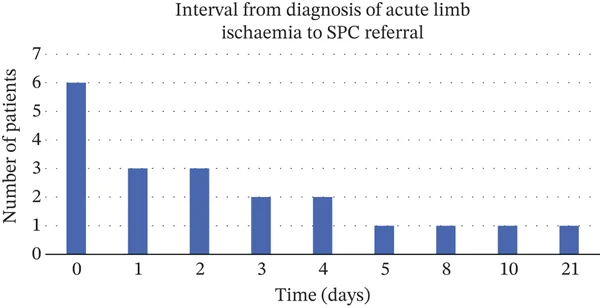
Referral timing.

**Figure 8:**
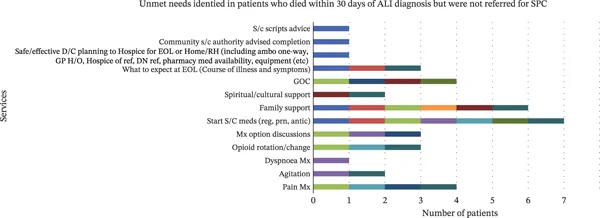
Potential SPC support.

Referrals ranged from specific requests, for example, ‘for pain management’ to broad requests of ‘please see for end‐of‐life (EOL) management’. Those referred with specific requests received additional services from the SPC team. The majority (80%) of referrals for SPC involved EOL management. The other 20% were for symptom management, in particular pain. Services provided by the SPC team are listed in Table 10.

**Table 10:** SPC services provided.

Services provided by the SPC team	Number of patients
Pain management	100%
Opioid change to manage pain	80%
CSCI initiation/administration/management	75%
Family support	75%
Safe discharge planning	65%
Goals of care discussions	55%
EOL location discussions	50%
Subcutaneous prescription advice/completion and advocating for appropriate place of symptom/EOL management	40%
Dyspnoea and agitation management	35%
What to expect at EOL and spiritual/cultural support	30%
Advocacy for EOL funding; bladder management; initiating local EOL pathway; mouthcares	20%
Medication reconciliation	15%
Advocacy for carer training; care after death; advice on switching from PCA to CSCI administration of medications	10%

Eighteen patients (90%) referred for SPC were for conservative management. The SPC team was involved in the decision for conservative management for three (15%) of these patients. The surgical team decided nine (45%) were not suitable for surgery, and eight (40%) were offered surgery but either chose comfort cares, or deteriorated and were no longer surgical candidates.

Two patients referred for SPC had had surgical revascularisation. The first suffered a cardiac arrest during the procedure requiring cardiopulmonary resuscitation (CPR) followed by continued postoperative limb ischaemia. The second had bilateral bypass surgery but was found to have nonviable legs 24 h postoperative. Both were referred when deemed too unstable for further surgery.

## 4. Discussion

Acute limb ischaemia is a vascular emergency associated with significant morbidity and mortality [1, 4]. This study found 30‐day mortality of 19% and 1‐year mortality of 31%, similar to that reported by previous studies [4–6, 18, 19]. Despite this high mortality, SPC was underutilised with many referrals made in the last days of life when earlier referrals would have improved symptom control and maximised quality of life. As in previous reports [13–16], this study highlighted the need to identify ALI patients with a poor prognosis at diagnosis to optimise treatment options and prompt referral for SPC.

### 4.1. Prognostic Markers

Research on prognostic factors in patients with ALI has been limited to date and identified age, gender, Rutherford ≥ IIb, renal impairment, anaemia, atrial fibrillation and history of coronary artery disease as possibilities [18, 19]. This study found four independent predictors of increased mortality: lactate > 2.2 mmol/L, albumin < 27 g/L, Rutherford Stage III ischaemia and baseline AKPS ≤ 50 or ≤ 40 at ALI diagnosis.

Despite the small cohort and incomplete dataset, low *p* values strongly supported the prognostic value of elevated lactate and reduced albumin. Cut‐off levels were determined, above and below which, respectively, mortality increased significantly. Previous studies have not investigated lactate or albumin levels as prognostic indicators or, as a result, defined cut‐off levels for these biomarkers.

Using documented AKPS scores to quantify ALI patients′ functional status and its relationship to outcome, this study corroborated previous research [20–22] that recommended functional status be used as a prognostic and treatment optimisation tool, and prompt for SPC referral. AKPS scores (both baseline and at ALI diagnosis) were found to be independent predictors of mortality, with mortality risk reduced by approximately 30%–40% for every 10‐point increase in AKPS. An 80% increased risk of mortality was seen with a baseline AKPS ≤ 50 and ≤ 40 at ALI diagnosis, which represent values of significance when using the AKPS tool in this patient population. Although, both AKPS scores offer prognostic information, the latter is likely to be more clinically relevant and practical for use by the admitting team.

Utilising the Rutherford classification to group patients by ALI severity, those with Rutherford Stage III ischaemia had an almost 10‐fold increased risk of death compared with those with Rutherford Stage I. Kulzeic and Acosta [19] and Tan et al. [23] reported similar findings and suggested that Rutherford Stages IIb and III are useful prognostic indicators.

Although further research is needed to validate this study′s findings, the four prognostic factors it found may be useful in the development of a risk stratification tool to identify ALI patients with poor prognosis, help guide treatment decisions and prompt earlier referral for SPC when appropriate.

### 4.2. SPC Referral

Surgical patients are less likely to receive SPC consultation than those under medical specialty services [13, 14], possibly because there has been more research on the benefits of SPC for the latter. This study confirmed previous reports of underutilisation of SPC for ALI patients with a 14% referral rate and average of 13 days between referral and death [15]. Mcintosh et al. [24] found that vascular surgeons did not usually seek SPC support until the patient was actively dying despite an expectation earlier in their disease trajectory that they were unlikely live for more than 12 months.

In this study patient cohort, 60% of SPC referrals were made within 2 days of ALI diagnosis. The remainder were between 3 days and 3 weeks post diagnosis. Of the eight patients referred for SPC more than 2 days after ALI diagnosis, five (62%) were identified as having inadequately addressed needs that could have benefitted from earlier SPC input. In particular, the patient who was not referred for 3 weeks had had considerable, unmet pain issues during that period that had been documented daily by nursing staff.

Our study found the SPC team was only involved in treatment option discussions for three (15%) of the referred patients although almost half of them and/or their families had to decide whether to opt for surgery or conservative care. The SPC team could have helped by explaining conservative care and offering support and perspective when they made treatment decisions.

ALI patients referred for SPC received a range of services, often in excess of those requested. Realising the need to increase awareness and encourage referrals, the SPC team has developed literature for other specialties describing what services they can provide for patients.

## 5. Conclusion

SPC is underutilised in patients with ALI. The four independent predictors of mortality found in this study (lactate > 2.2 mmol//L, albumin < 27 g/L, Rutherford Stage III ischaemia and AKPS ≤ 50 at baseline or ≤ 40 at ALI diagnosis) may be useful in the development of a risk stratification tool to identify high‐risk patients, optimise treatment options and prompt earlier referral for SPC. Future prospective studies are needed to validate the sensitivity and specificity of these prognostic factors and their combined use as a risk stratification tool. In the meantime, it is strongly recommended that patients exhibiting these risk factors are considered for early referral for SPC.

### 5.1. Limitations

This was a single centre, retrospective review reliant on the completeness and accuracy of information previously recorded in patients′ clinical records by physicians, nurses and allied health professionals. Together with one third of the patient cohort missing data for biomarkers, the generalisability of the results is limited. A prospective trial would provide full and accurate data, and enable validation of the identified prognostic factors.

## Funding

This research is supported by the Waikato Hospital Research Committee and the Waikato Hospital Te Puna Oranga Māori Research Review Committee.

## Conflicts of Interest

The authors declare no conflicts of interest.

## Data Availability

The data that support the findings of this study are available on request from the corresponding author. The data are not publicly available due to privacy or ethical restrictions.
